# Impact of Anthropogenic Activities on Underwater Noise Pollution in Venice

**DOI:** 10.1007/s11270-022-05653-2

**Published:** 2022-06-07

**Authors:** Jacopo Boaga, Lapo Boschi

**Affiliations:** 1 Dipartimento di Geoscienze, Università degli Studi di Padova, Padua, Italy; 2 Sorbonne Université, CNRS, INSU, Institut des Sciences de la Terre de Paris, ISTeP UMR 7193, F-75005 Paris, France; 3 Istituto Nazionale di Geofisica e Vulcanologia, Bologna, Italy

**Keywords:** Cultural noise, Motorboat noise, Underwater noise, Venice historic center

## Abstract

We illustrate the implementation and results of a field experiment, consisting of recording continuous signal from a hydrophone 3 m deep in the Venetian lagoon. We simultaneously recorded audio signal through a microphone placed on a nearby pier. We investigate the potential of this simple instrumental setup to explore the small touristic boat traffic contribution to the underwater noise. The ultimate goal of our work is to contribute to quantifying underwater noise pollution due to motorboat passages and its impact on the ecosystem. Efforts such as ours should help to identify measures that could diminish noise pollution, focusing specifically on the aspects that are most disruptive to underwater life. After this preliminary test, more work can be planned, involving the deployment of a larger network of similar instruments around the lagoon. At this point, we can conclude that (i) our instruments are sensitive enough to detect motorboats and identify some of their characteristics; (ii) the area of interest is characterized by a large (approx. 20 dB) day/night difference in ambient noise; and (iii) the historic center of Venice and its immediate surroundings are particularly noisy, in comparison to other similarly studied locations.

## Introduction

**Fig. 1:**
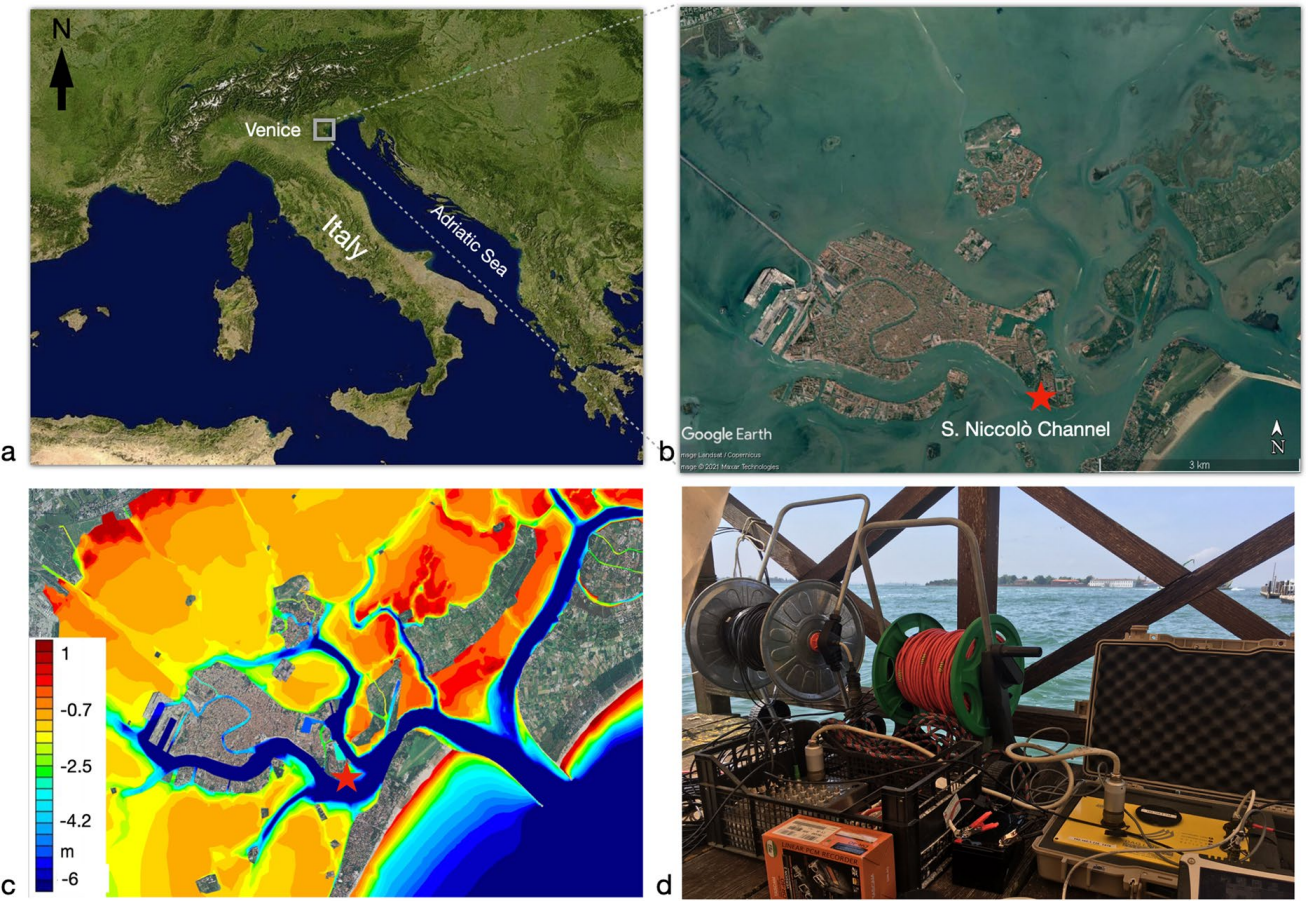
Experimental setup. (**a**) Map of Italy (Google Earth image); (**b**) map of the historic center of Venice and the location of our deployment (red star), along the S. Niccolò channel (Google Earth image); (**c**) bathymetry (http://dx.doi.org/10.6084/m9.figshare.1293558 (open data CC-BY 4.0), mod.); (**d**) photograph of our recording equipment, on the pier of F. Morosini high school

The purpose of the experiment presented in this paper was to determine whether a simple instrument setup, typically employed in seismic exploration, could provide useful information on the soundscape of a busy harbor area, and in particular on the noise produced by motorboat traffic and, possibly, other anthropic activities. We accordingly deployed a set of hydrophones on the bottom of one of the channels bordering the city of Venice, near a convenient observation point on the premises of the Navy School “F. Morosini”, island of Sant’Elena (see Fig. 1). We complemented this setup with a portable audio recorder.

There are important reasons today for studying marine soundscapes. Human activities are well known to have a profound and growing impact on biodiversity and on the environment as a whole (Frisk, 2012). In March 2021, the new “Horizon Europe” strategic plan of the European Union identified “Healthy oceans, seas, coastal and inland waters” as one of its five “mission areas” European Commission (2021). One important aspect of our impact on wildlife is noise pollution, whether in the form of elastic (e.g., seismic noise from farms, oil and gas rigs) or acoustic waves (seismic surveys, shipping). It has been proved that marine wildlife is affected by shipping traffic, seismic surveys, and other anthropic activities (e.g., Kedar et al. 2008): the frequency range of anthropic and biological signals overlap, resulting in disruption of animal behaviors, such as masking communication or hindering larval settlement.

The environment of Venice historic center is particularly fragile and threatened both by climate change and the growing impact of mass tourism. Climate change impacts sea level rise, and as such has led to the design and implementation of mitigation policies (Del Bello 2018; Zanchettin et al. 2021); mass tourism, on the other hand, has scarcely been considered as an environmental issue by policy-makers. Nevertheless, growing cruise-ship traffic and the growing demand for tourist transportation are among the main causes of water and air pollution in Venice (Contini et al. 2011; Scarpa et al. 2019). The same activities impact significantly the noise level of the area, both in air (Agenzia Regionale per la Prevenzione e Protezione Ambientale Regione Veneto and Comune di Venezia 2002) and underwater. Bolgan et al. (2016) measured underwater noise at the border of Venice city center for a short diurnal period, highlighting the relevant noise level increase (up to 138 dB with respect to 1 µPa) associated with the passage of large ships. Tegowski et al. (2019) studied the seasonal variations of underwater noise in Venice, showing that summer is considerably noisier than winter, owing mainly to mass tourism and fishery. Picciulin et al. (2021) proved that motorboats and anthropic activities in Venice lagoon and tidal inlets significantly affect the life and behavior of fish. It is known that motorboats generate noise in a frequency range that can bother several coastal fish species, such as *Chromis chromis* and *Gobius cruentatus* (Codarin et al. 2009), *Pimephales promelas* (Scholik and Yan 2002), goldfish (Smith et al. 2004); largemouth bass (Graham and Cooke 2008); damselfish (McCloskey et al. 2020; Holmes et al. 2017) and cetaceans (Marley et al. 2017). In particular, intense motorboat activities affect the reproductive behavior of *Sciaena umbra* (Picciulin et al. 2021), a widespread species in the Venice lagoon environment. The impact of anthropic activities on the underwater environment of Venice Lagoon was confirmed by Braga et al. (2020), who observed the effects of the recent lockdown imposed by the COVID-19 pandemic. The associated reduction in boat and ship traffic led to observable changes in water transparency, and to the attenuation of noise pollution.

In the following, we contribute to quantifying the underwater noise level floor of the Venice city center due to motorboat traffic and other anthropic activities, comparing the soundscape at rush hour vs. night-time. We focus on the low-frequency range induced by the passages of several types of motorboats. Our results show that the Venice historic center is constantly subjected to high underwater noise, if compared to other coastal environments (Kaplan and Mooney 2015; Merchant et al. 2016; Pieretti et al. 2020). Further work is needed to better identify specific noise sources, with the goal of guiding future mitigation policies.

## Material and Methods: Experimental Setup and Observations

On June 24, 2021, we deployed three ETL-tech P-44A hydrophones in the S. Nicolò canal, some 20 m offshore from the Island of S. Elena, in Venice, Italy (Fig. 1). The area is characterized by relatively heavy motorboat traffic and can be considered as representative of the channels bordering the center of Venice. We additionally placed one TASCAM audio recorder, operating its built-in stereo microphone, on the pier where all our recording equipment was installed. The hydrophones were dropped into approximately 3 m deep water. The three hydrophones were connected to one another by cable, lying on the sea bottom and spaced about 0.5 m from one another. Their frequency response is flat above 8 Hz. They were operated through a 12-channel DAQ LINK III recorder, which is a portable seismograph with a 24-bit digitizer, 112 dB dynamic range, noise floor $< 0.2$ µV RMS (@ 2ms), integrated anti-aliasing filter and GPS clock.

Our experiment consisted of simultaneously recording hydrophone and audio data for 80 min starting at 11:10 am, and again hydrophone data for 60 min starting at 9:30 pm, i.e., in the late evening when traffic is usually reduced. In both cases, we set the sampling rate at 1000 Hz. During the evening recording session, relatively strong winds (approx. 20 km/h) resulted in a very noisy audio recording, that we soon decided to interrupt: for this reason, no audio data of the evening session are available.

**Fig. 2:**
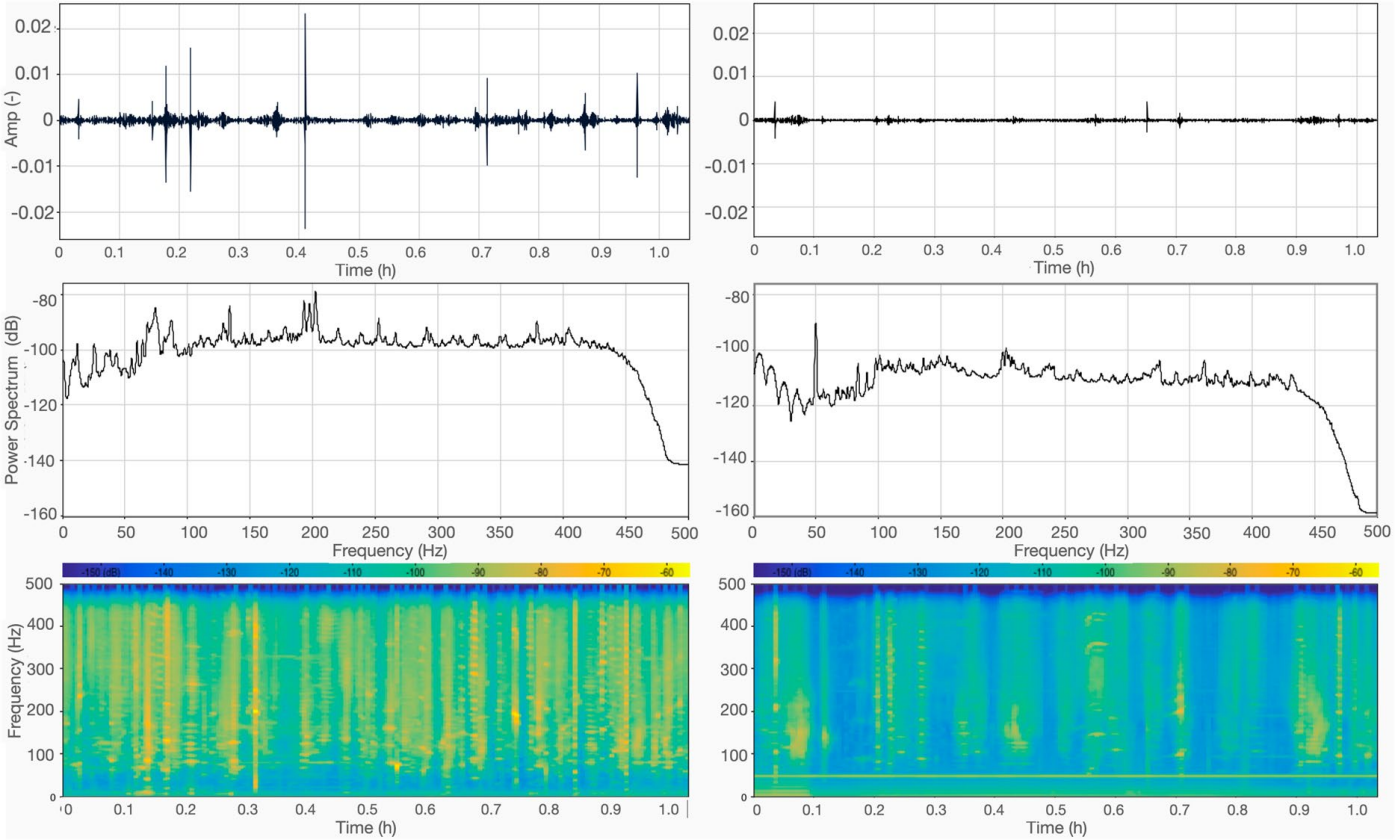
Day-time (left) vs. night-time (right) records. Top: time series; middle: power spectra of the Fourier transforms; bottom: spectrograms of the signal. The overall drop in noise level can be appreciated in all three visualizations of the data

Day-time and night-time hydrophone observations are summarized in Fig. 2. No significative differences are observed between the signals recorded by the three sensors that we had deployed, and in the interest of brevity, here and throughout the rest of the paper, we are only showing data from one hydrophone.

A significant difference in the overall energy of the recorded signal is immediately apparent in Fig. 2, with the night-time much quieter than day-time (roughly 10 to 20 dB on average). This is not surprising, but confirms that the majority of underwater noise in the Venetian lagoon is the result of anthropic activities, at least in the instrumented area. The power spectra are relatively flat at most frequencies. The 50 Hz peak in the night-time recording can be interpreted as interference from the electrical illumination of the pier where our recording gear was located.

### Footprints of Different Motorboats

**Fig. 3:**
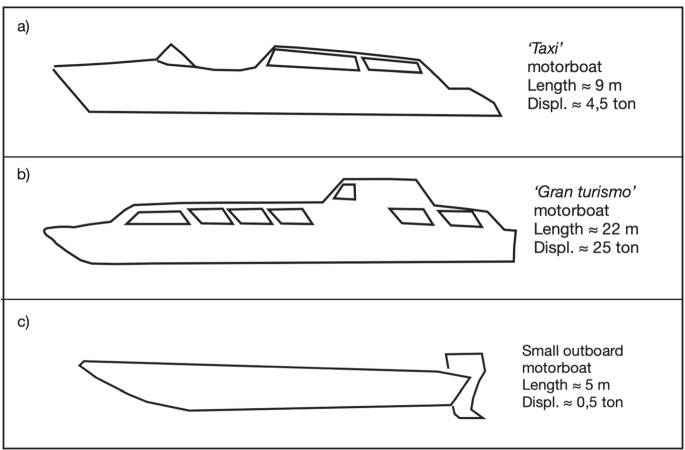
Sketches of the three most typical motorboats used in the Venice area: “taxi” (**a**), “Gran Turismo” (**b**) and small outboard motorboard (**c**). Typical total length in m and displacement in tons are indicated in each panel. (**a**) and (**b**) are diesel-powered, while (**c**) runs on petrol

We next investigate in some detail individual “noise events”, via the analysis of simultaneous hydrophone and audio recordings during the passage of motorboats. We pick three examples, each associated with one of the common motorboats sketched in Fig. 3. During the day-time recording session, sea state was $<2$ on the Douglas scale, and wind speed $< 10$ km/h.

**Fig. 4:**
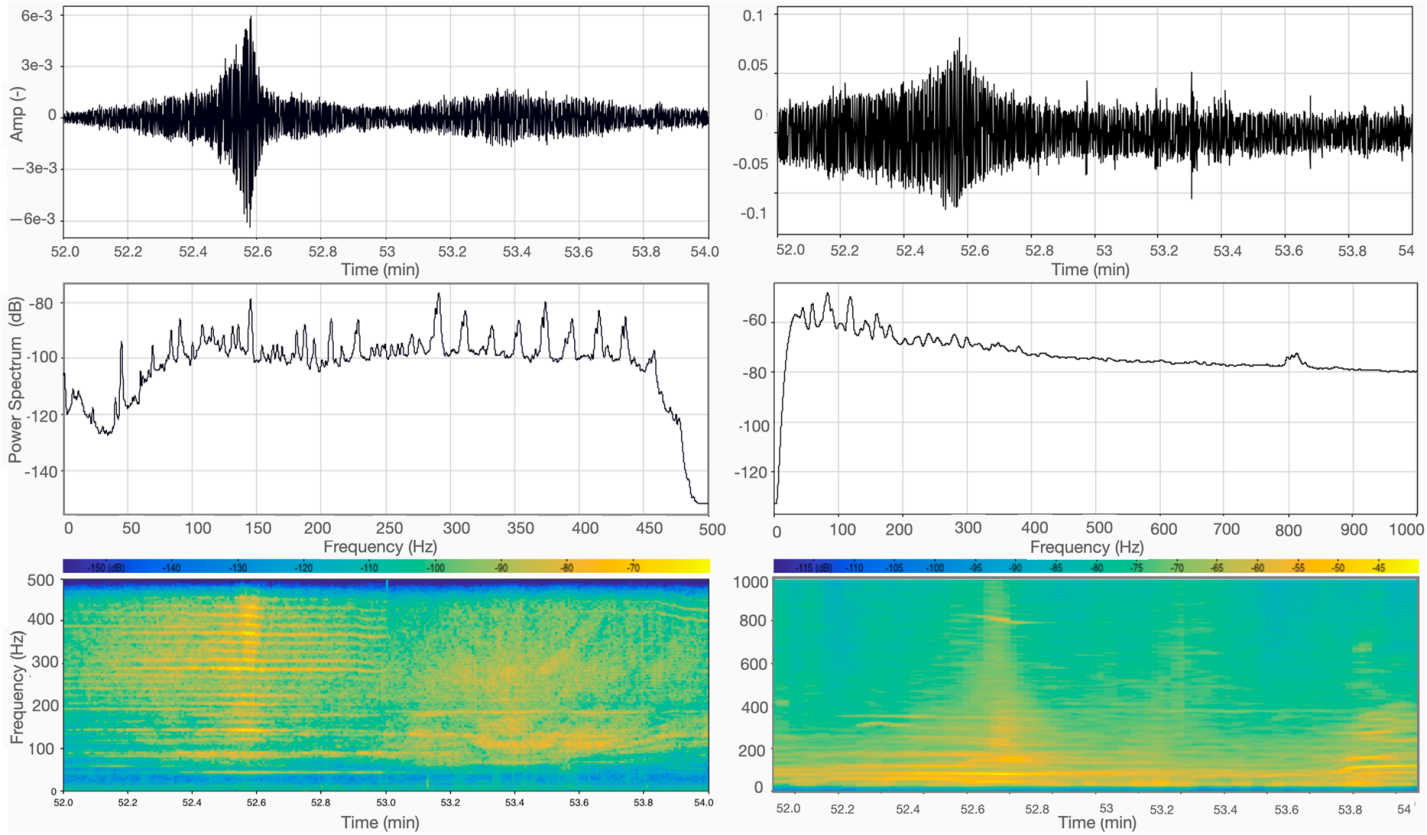
2-minute hydrophone (left) and audio (right) recordings, taken 52 min after the beginning of the day-time recording session during the passage of a taxi boat (Fig. 3a). Time series (top), power spectrum (middle) and spectrogram (bottom) are all shown. Both spectrograms were obtained using 938-ms-long temporal windows with $50\%$ overlap, and frequency resolution bandwidth of 4 Hz. The amplitude scales of hydrophone and audio “raw” data shown here are normalized differently and cannot be compared quantitatively; hydrophone data are converted to SPL in the following

The three types of boat have different underwater and audio noise signals. We show in Fig. 4 the footprint of the passage of a taxi boat (model *a*) passing at moderate velocity (approx. 15 knots) close to our sensors (roughly 50 m). During the time we spent on the pier, we repeatedly saw such boats passing relatively close (from 30 to 50 m) to the location of our receivers.

Both the hydrophone and microphone appear to be sensitive to this event, with hydrophone data showing multiple sharp peaks that presumably reflect the power and make of the engine (e.g. Holmes et al. 2017; Tegowski et al. 2019). The intensity peak in the time series occurs at the same time as the frequency shift in the spectrogram, and we interpret the latter as an instance of the Doppler effect: compare, e.g., with Fig. 4b of Trabattoni et al. (2020). Audio data show qualitatively similar spectral peaks at relatively low frequencies (below 500 Hz), with one isolated peak at 800 Hz.

**Fig. 5:**
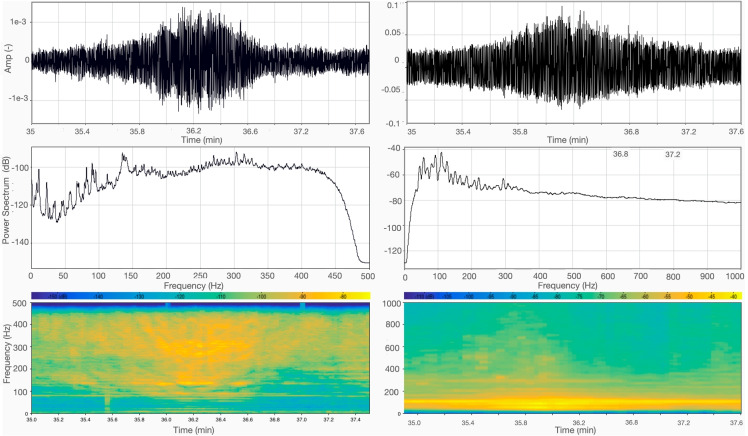
Same as Fig. 4, but data recorded 35 min after the beginning of the day-time session during the passage of a “Gran Turismo” boat (Fig. 3b)

Figure 5 shows the passage of a typical tourist transportation boat (Fig. 3b), passing at a larger distance (roughly 200 m) from our receivers at moderate velocity (approx. 10 knots). This, again, corresponds to our visual observation of boat traffic from the pier.

Again, both hydrophone and microphone are sensitive to this event, and, in particular, the frequency peaks in hydrophone data are distributed over a much broader range than those of Fig. 4. A few, relatively less energetic but well-spaced lower-frequency peaks are visible in the hydrophone power spectrum; higher-frequency peaks are numerous and very closely spaced, so that the lower-frequency ones might be most useful in an effort to identify the nature of the event (technical characteristics of the boat, etc.)

**Fig. 6:**
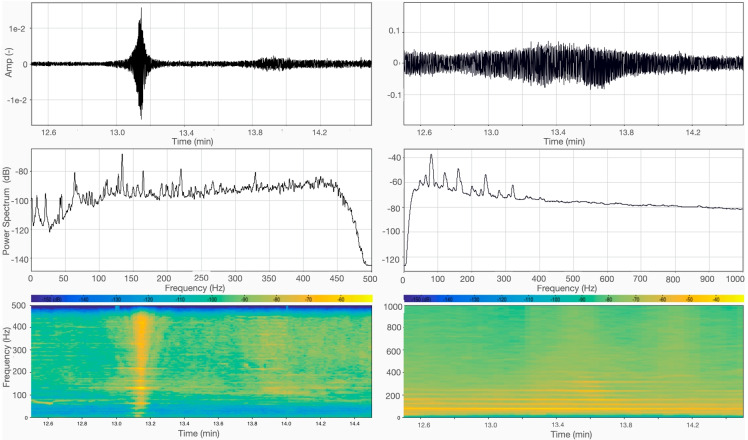
Same as Fig. 4, but data recorded 12 min after the beginning of the day-time session during the passage of a small outboard motorboat (Fig. 3c)

Figure 6 shows the passage of a small outboard boat roughly 30 m from our pier at an estimated velocity of around 15 knots. These boat passages were frequent during our day-time recordings. Compared to other boats, small motorboats are characterized by relatively high power in the high-frequency range, while in the microphone recording multiple sharp peaks at low frequencies are detected (below 300 Hz).

Numerous events, with characteristics similar to those illustrated in Figs. 4, 5 and 6 are observed throughout our data set. We chose to focus on the described popular types of motorboat, that are the main responsible of the city waters swell.

### Analysis of “Background” Signal

As seen from Fig. 2, while less intense than during the day, traffic was still non negligible during our night-time recording session. To evaluate the soundscape of the Venice lagoon in the absence of nearby motorboat traffic, we selected 1 min of recording during which no traces of motorboat passages are visible and no boats were detected in the surrounding, to be compared with a typical passage of motorboat.

**Fig. 7:**
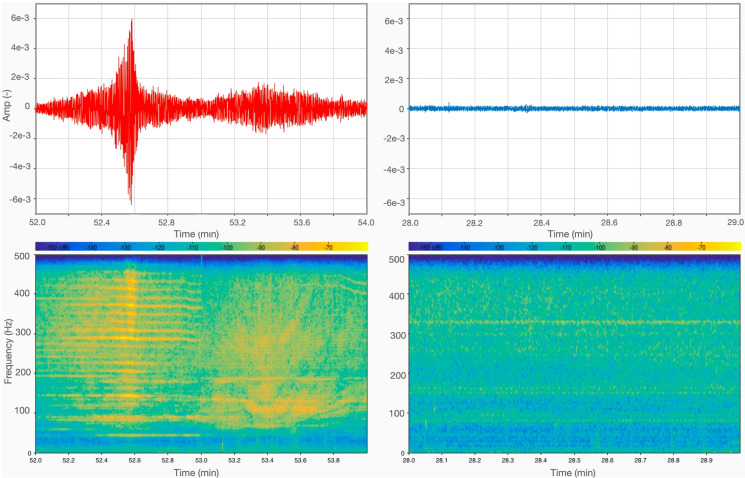
Noise vs. silence: passage of a taxi boat during day-time (left panels), compared with 1 min of signal, recorded by the hydrophone 36 min after the beginning of the night-time recording session when no events that can be attributed to anthropic activities are recognized (right). Top: time series, bottom; spectrogram of the signal. The A/C electric noise at 50 Hz was removed from night-time data through a notch filter

**Fig. 8:**
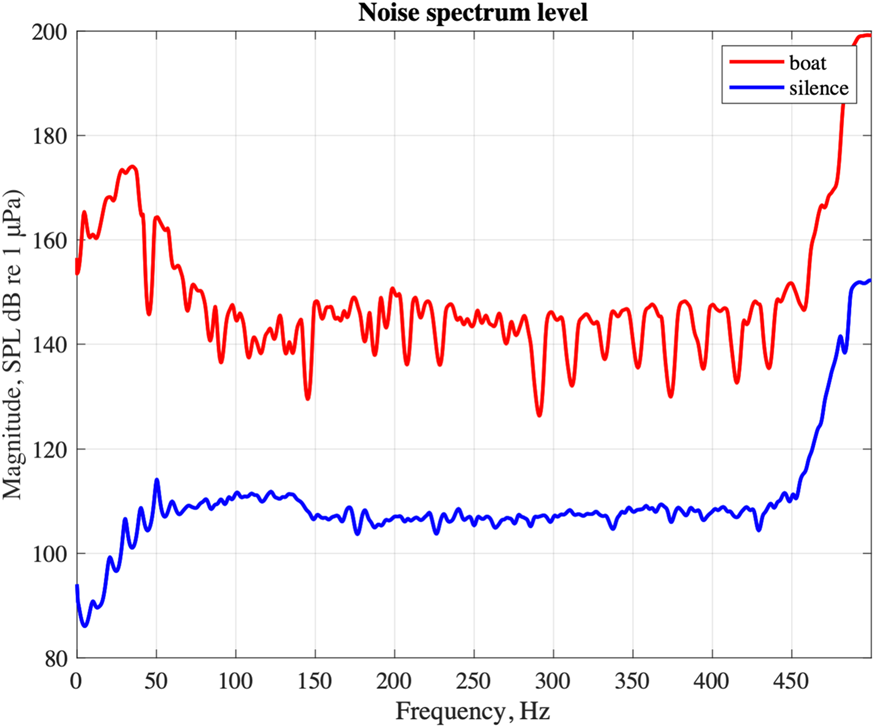
Sound pressure level magnitude during a boat passage (red line) vs. during the quietest night period recorded (blue line)

We show in Fig. 8 the power spectra obtained from the time series of Fig. 7, quantifying the noise produced by a typical taxi boat in comparison with a reference, “quiet” noise level (night-time, no discernible nearby boat passages). The spectrum is provided here in terms of sound pressure level magnitude, expressed in decibels relative to 1 µPa, which should facilitate comparison with the results of other studies in the literature. Based on the limited data available to us at this point, we estimate a difference of at least 30 dB between overall noise level during a typical, “silent” day-time recording, and the quietest night-time intervals that we have been able to record.

## Discussion and Conclusions

Our pilot experiment has shown that a small set of instruments, originally designed for seismic exploration purposes, have significant sensitivity to underwater acoustic signals generated by motorboat traffic. The details of the spectrograms and power spectra that we have obtained (see the examples in Figs. 4 and 5) suggest that certain technical characteristics of a motorboat might be determined from such data alone. Further work is needed to substantiate this speculation. We note, however, that boat engines are known to excite discrete sets of “tonal” peaks, at least qualitatively similar to what we have recorded, related to the propeller type, the number of revolutions and the hull shape (e.g., Carlton 2018).

We find that common motorboats used in Venice, such as the “Gran Turismo” of Fig. 3b, generate significant underwater noise in the frequency range 200–300 Hz that is relevant, e.g., to the *Sciaena Umbra* (Picciulin et al. 2021). In the same frequency range, smaller motorboats are much quieter (-30 dB), according to both audio and hydrophone data. We surmise that policy-makers should take account of observations such as ours, in view of implementing regulations (involving, e.g., the values of boat engine parameters noted above) that should reduce disruption of animal life, with a reasonable impact on human activities.

We have also evaluated the background noise level, in the absence of nearby boat traffic, both in day- and night-time (Fig. 7). At most frequencies, we find noise to be around 130 dB during the day in the absence of traffic; this is amplified to about 150 dB at the passage of a motorboat, and drops to 110 dB at night, again in the absence of traffic. After compiling a selection (certainly not complete, but hopefully sufficiently representative) of related literature, we are able to compare our findings to those of other authors, who studied the same or other, more or less similar areas.

**Table 1:** Selected observations of underwater background noise levels, given in dB with respect to 1 µPa

Bibliography reference	Area of study	Noise level	Time of recording
Bolgan et al. (2016)	Venice, Italy	120–130	Day-time only
Cafaro et al. (2018)	Civitavecchia, Italy	106	Day-time only
Codarin et al. (2009)	Trieste, Italy	108	Day-time only
Holmes et al. (2017)	Lizard, Australia	80	Day-time only
Kaplan and Mooney (2015)	Virgin Islands, USA	90	Day- and night-time
Picciulin et al. (2021)	Trieste, Italy	100	Day-time only
Pieretti et al. (2020)	Naples, Italy	124	Day-time only
Merchant et al. (2016)	UK	90	Day-time only
Tegowski et al. (2019)	Venice, Italy	115	All season average
Soares et al. (2020)	Ria Formosa, Portugal	100	Day- and night-time
This study	Venice, Italy	145, 110	Day- and night-time

Table 1 confirms that the underwater soundscape of Venice seems to be relatively very noisy, even in comparison with busy ports such as Naples and Trieste. Our estimate of day-time background noise is comparable with the observation made by Bolgan et al. (2016) also in the Venice lagoon. Night-time background noise, as we observe it, drops to below the average (day and night) value observed, again in the Venice lagoon, by Tegowski et al. (2019). We surmise that, besides motorboat traffic, this high level of noise might be explained in terms of the wide range of anthropic activities taking place in the city. A recent paper by our team (Poli et al. 2021) has shown that the 2020 pandemics-related lockdown resulted in a reduction of ambient seismic noise more drastic in Florence than in any other investigated Italian city. The economy of Florence, like that of Venice, is largely based on tourism: we accordingly infer from our findings and those of Poli et al. (2021) that activities related to tourism could result in particularly significant levels of noise pollution.

In summary, our study confirms that boat traffic contributes a great deal to the noise in the Venice channel. Several different anthropogenic activities (including, but not limited to motorboat traffic) have probably a constant impact on the soundscape of the Venice lagoon, an environmentally fragile area with, in addition, significant cultural value. In agreement with the European Union Marine Strategy Framework Directive (European Commission 2008), and Horizon Europe missions (European Commission 2021), it will be important to monitor the future evolution of background noise in the lagoon, and determine how disruptive it might be for underwater life.
